# Supplementary material for: Attenuating Colorectal Cancer Using Nine Cultivars of Australian Lupin Seeds: Apoptosis Induction Triggered by Mitochondrial Reactive Oxygen Species Generation and Caspases-3/7 Activation
Source: Cells. 2023 Oct 31;12(21):2557. doi: 10.3390/cells12212557 (PMC10647522; doi:10.3390/cells12212557)
Supplement: Supplementary file 1 [file cells-12-02557-s001.zip › cells-2653996-supplementary.pdf]

cells

MDPI

Supplementary Materials

Attenuating Colorectal Cancer Using Nine Cultivars of Australian Lupin Seeds: Apoptosis Induction Triggered by Mitochondrial Reactive Oxygen Species Generation and Caspases-3/7 Activation

Kishor Mazumder [1,2,*], Asma Aktar [3], Sujatha Ramasamy [4], Biswajit Biswas [1,5], Philip G. Kerr [6] and Christopher Blanchard [6]

[1] Department of Pharmacy, Jashore University of Science and Technology, Jashore 7408, Bangladesh
[2] School of Optometry and Vision Science, UNSW Medicine, University of New South Wales (UNSW), Sydney, NSW 2052, Australia
[3] Department of Pharmacy, Dhaka International University, Dhaka 1212, Bangladesh
[4] Institute of Biological Sciences, Faculty of Science, Universiti Malaya, Kuala Lumpur 50603, Malaysia
[5] Institute for Molecular Bioscience, Queensland University, Brisbane, QLD 4072, Australia
[6] School of Biomedical Sciences and Graham Centre for Agricultural Innovation, Charles Sturt University, Boorooma St., Wagga Wagga, NSW 2650, Australia
* Correspondence: kmazumder@just.edu.bd or k.mazumder@unsw.edu.au

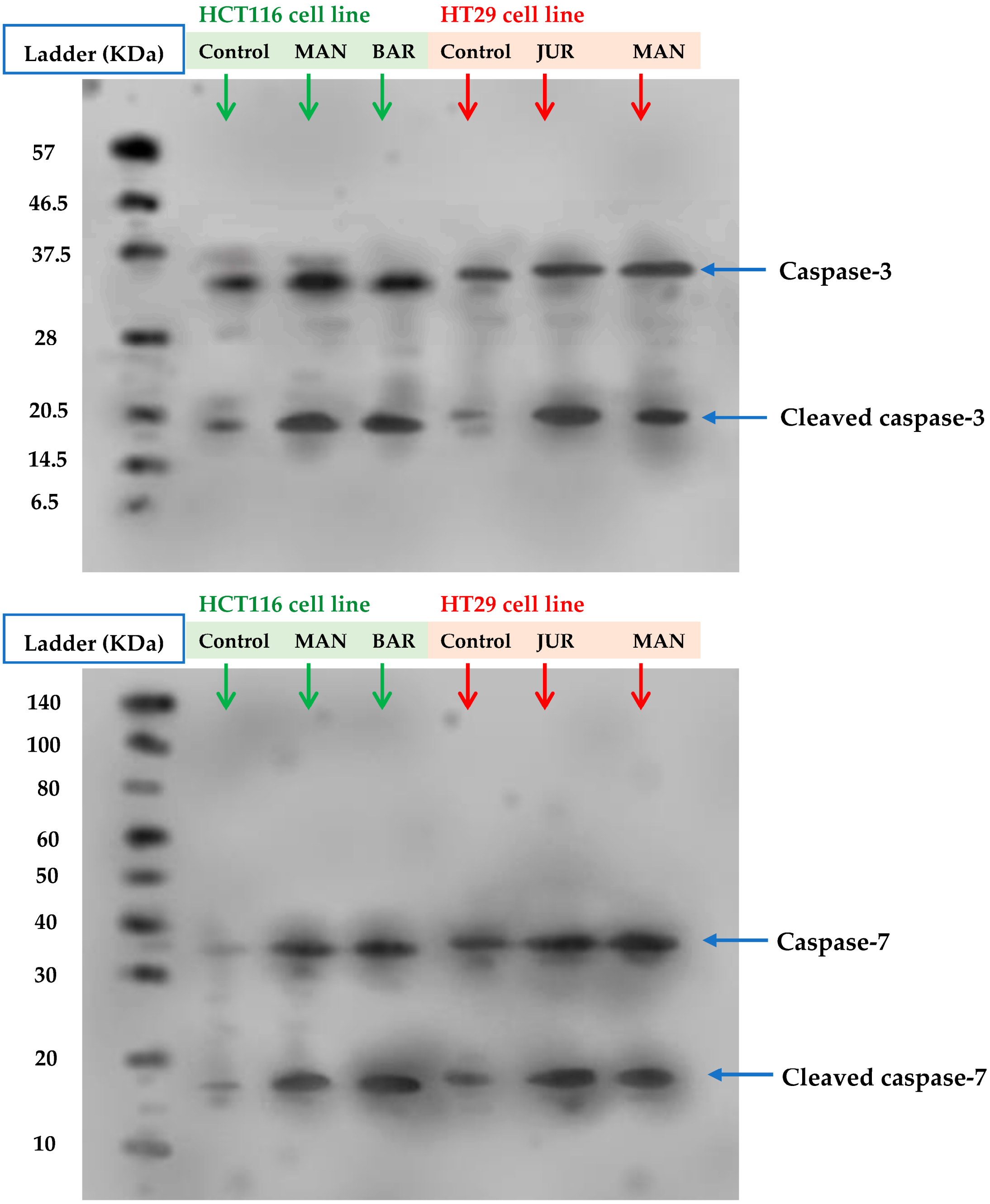

Figure S1. Full length images of Western blots in caspase-3/7 detection assay. MAN: Mandelup, BAR: Barlock, and JUR: Jurien.
